# Clinical results of combined vemurafenib and tumor-infiltrating lymphocyte therapy for metastatic melanoma

**DOI:** 10.1186/2051-1426-3-S2-P49

**Published:** 2015-11-04

**Authors:** Amod Sarnaik, MacLean Hall, John Mullinax, Erica Royster, Allison Richards, Georgina Crago, Jonathan Zager, Sondak Vernon, Jeffrey Weber, Shari Pilon-Thomas

**Affiliations:** 1 H. Lee Moffitt Cancer Center, Tampa, FL, USA; 2 H. Lee Moffitt Cancer Center & Research Institute, Tampa, FL, USA

## Background

We previously established the feasibility of Tumor-Infiltrating Lymphocyte (TIL) cell therapy for unresectable metastatic melanoma at our institution[1]. This involves resection of tumor from patients for *in vitro* TIL generation. Upon satisfactory TIL growth, patients are rendered lymphopenic with non-myeloablative chemotherapy and receive adoptive cell therapy with TIL followed by high dose Interleukin-2. We observed a 38% response rate, with all responses ongoing to date (range, 34 to >54 months). However, 35% of patients who underwent tumor harvest dropped out prior to TIL transfer, primarily due to progression of disease. This limitation may be addressed by inclusion of additional treatment prior to TIL transfer. Therefore, we combined vemurafenib with TIL therapy in a clinical trial for patients with unresectable, BRAF V600-mutated metastatic melanoma.

## Methods

14 patients with unresectable, BRAF V600-mutated metastatic melanoma were accrued to an Institutional Review Board-approved trial (NCT01659151). All pts had >1 cm^3^ of soft tissue or nodal metastases resected for TIL generation leaving residual measurable disease. Vemurafenib started the day after TIL harvest, was stopped prior to TIL therapy, and then resumed after recovery from TIL therapy. Clinical responses after TIL transfer were evaluated by RECIST 1.1 criteria.

## Results

Thirteen of 14 (93%) accrued patients received a median of 5.1e10 TIL (9e9 – 8.6e10 cells). One patient was not treated due to inadequate TIL growth. No patient dropped out due to disease progression before receiving TIL. Of 12 evaluable patients, there were 5 responders (42% - 1 complete, 4 partial responses) and 7 non-responders (58% - 2 stable disease, 5 progressive disease). Median progression-free survival was not reached at a median follow-up of 364 days (range 104 – 910 days). There were no treatment-related deaths.

## Conclusions

The combination of vemurafenib with adoptive TIL therapy for unresectable metastatic melanoma is feasible and has the potential to reduce disease progression prior to TIL transfer. This approach serves as a model for future efforts to combine TIL with newer therapies including inhibitors of BRAF/MEK, PD-1/PD-L1 blocking antibodies, and other emerging immune checkpoint inhibitors.
